# MLGO: phylogeny reconstruction and ancestral inference from gene-order data

**DOI:** 10.1186/s12859-014-0354-6

**Published:** 2014-11-08

**Authors:** Fei Hu, Yu Lin, Jijun Tang

**Affiliations:** Tianjin Key Laboratory of Cognitive Computing and Application, Tianjin University, Tianjin, 300072 China; Department of Computer Science and Engineering, University of South Carolina, Columbia, 29208 SC USA; Department of Computer Science and Engineering, University of California, San Diego, 92093 La Jolla CA USA

**Keywords:** Phylogeny reconstruction, Ancestral inference, Genome rearrangement, Maximum likelihood

## Abstract

**Background:**

The rapid accumulation of whole-genome data has renewed interest in the study of using gene-order data for phylogenetic analyses and ancestral reconstruction. Current software and web servers typically do not support duplication and loss events along with rearrangements.

**Results:**

MLGO (Maximum Likelihood for Gene-Order Analysis) is a web tool for the reconstruction of phylogeny and/or ancestral genomes from gene-order data. MLGO is based on likelihood computation and shows advantages over existing methods in terms of accuracy, scalability and flexibility.

**Conclusions:**

To the best of our knowledge, it is the first web tool for analysis of large-scale genomic changes including not only rearrangements but also gene insertions, deletions and duplications. The web tool is available from http://www.geneorder.org/server.php.

## Background

As whole genomes are sequenced at increasing rates, using gene-order data^a^ for phylogenetic analyses and ancestral reconstruction is attracting increasing interest. Comparative genomics, evolutionary biology, and cancer research all require tools to elucidate the history and consequences of the large-scale genomic changes, such as rearrangements, duplications, losses. However, using gene-order data has proved far more challenging than using sequence data and numerous problems plague existing methods: oversimplified models, poor accuracy, poor scaling, lack of robustness, lack of statistical assessment, etc.

Genome rearrangement operations change the ordering of genes on chromosomes. An *inversion* operation (also called *reversal*) reverses both the order and orientation of a segment of a chromosome. A *transposition* is an operation that swaps two adjacent segments of a chromosome. In case of multiple chromosomes, a *translocation* breaks a chromosome and reattaches a part to another chromosome, while a *fusion* joins two chromosomes and a *fission* splits one chromosome into two. Yancopoulos *et al.* [1] proposed a universal *double-cut-and-join* (DCJ) operation that accounts for all rearrangements used to date. None of these operations alter the gene content of genomes, whereas *deletions* (or *losses*) delete segments of (one or more) contiguous genes from a chromosome, while *insertions* introduce a segment of (one or more) contiguous genes from external sources into a chromosome. and *duplications* copies an existing segment within the genome and inserts into a chromosome. Finally, *whole genome duplication* (WGD) creates an additional copy of the entire genome of a species.

As phylogenies play a central role in biological research, over the past decade many methods were developed to reconstruct phylogenies from gene-order data. The first algorithm for phylogeny inference from gene-order data was BPAnalysis based on breakpoint distances [2]. Moret *et al.* [3] later extended this approach with GRAPPA by using inversion distances. While these methods were limited to unichromosomal genomes, Bourque and Pevzner [4] developed MGR to handle multichromosomal genomes. These approaches are parsimony-based: they solve the so-called Big Parsimony Problem (BPP) and all suffer from serious scalability issues. In contrast with parsimony-based methods, distance-based methods run in time polynomial in the number and size of genomes. Lin *et al.* [5] have demonstrated the accuracy and scalability of a distance-based method that uses NJ [6] and FastME [7] with an accurate distance estimator [8]. Instead of working directly with the evolutionary events of the model, one can also transform the problem into the familiar sequence-based reconstruction problem. Wang *et al.* [9] first proposed a parsimony-based approach, MPBE (Maximum Parsimony on Binary Encoding). Recently Hu *et al.* [10] developed MLBE, later refined by Lin *et al.* [11] with MLWD, both of which demonstrate that using maximum-likelihood approaches is the decisive factor in improving the modest accuracy of MPBE.

If the tree is fixed, then computing its parsimony score is known as the Small Parsimony Problem (SPP). Ancestral reconstruction has been studied through several optimization schemes for SPP on gene-order data—using adjacencies [12–15], using conserved intervals (Roci—Reconstruction of Conserved Intervals [16]), using multiple breakpoint graphs (MGRA [17]) and supporting whole-genome duplications [18,19], where continuous regions or complete ancestral genomes have been inferred.

Relatively few of these tools are offered through web servers. Lin *et al.* [20] had developed a web-server version of MGR with new heuristics to speed up the original MGR algorithm, but the site is no longer accessible. Both Roci and MGRA (for ancestral reconstruction only) are offered through web servers, but none can handle complex events such as gene insertions, deletions and duplications.

We present a new tool MLGO for the reconstruction of phylogeny and/or ancestral genomes from gene-order data. MLGO relies on two methods we have developed: MLWD [11] for phylogenetic reconstruction and PMAG+ [21] for ancestral genome reconstruction. Our tool takes the advantage of binary encoding on gene-order data, supports a fairly general model of genomic evolution (rearrangements plus duplications, insertions, and losses of genomic regions), and successfully accommodates itself into the framework of maximized likelihood. The results of extensive testing on both simulated and real data show that both MLWD and PMAG+ can achieve great performance, scalability and flexibility, suggesting MLGO a suitable tool for large-scale analysis of high-resolution data. Furthermore, MLGO is deployed as a web service, providing the first web tool that is suitable for large scale genomic analysis with a general model of evolution.

## Implementation

MLGO preprocesses the gene-order data, configures the transition model, reconstructs a phylogeny, and finally solves the SPP on that phylogeny.

### Terminology

Given a set of *n* genes labeled as {1,2,⋯,*n*}, gene-order data for a genome consists of lists of genes in the order in which they are placed along one or more chromosomes. Each gene is assigned with an orientation that is either positive, written *i*, or negative, written −*i*. Two genes *i* and *j* form an *adjacency* (*i*,*j*) if *i* is immediately followed by *j*, or, equivalently, −*j* is immediately followed by −*i*. If gene *k* lies at one end of a linear chromosome, we let *k* be adjacent to an extremity *o* to mark the beginning or ending of the chromosome, written as (*o*,*k*) or (*k*,*o*), and called *telomere*.

### Phylogeny reconstruction

The data preprocessing and the configuration of the transition model follow the approach of MLWD [11]. Each adjacency that appears at least once in the collection of input genomes corresponds to a unique character position in the sequence and the presence or absence of any of these adjacencies in a given genomes is coded by a 1 (presence) or a 0 (absence). Since our encodings are binary sequences, the parameters of the model are simply the transition probability from presence (1) to absence (0) and that from absence (0) to presence (1). Lin *et al.* [11] gave the following derivation for these parameters. A DCJ operation selects uniformly at random two adjacencies (or telomeres) and replaces them by two new adjacencies (or telomeres). Since a genome with *n* genes and *O*(1) chromosomes has *n*+*O*(1) adjacencies and telomeres, the transition probability from 1 to 0 is $\frac {2}{n+O(1)}$ under one DCJ operation; and since there are up to $2n+2\choose 2$ possible adjacencies and telomeres, the transition probability from 0 to 1 is $\frac {2}{2n^{2}+O(n)}$. Thus the transition from 0 to 1 is roughly 2*n* times less likely than that from 1 to 0. Despite the restrictive assumption that all DCJ operations are equally likely, this result is in line with the observed bias in transitions of adjacencies given by Sankoff and Blanchette [22]: the probability of breaking a given ancestral adjacency is high while that of creating a particular adjacency along several lineages is low (a version of homoplasy for adjacencies). Finally, the encoding adds characters and a transition probability for the presence or absence of each unique gene. Due to duplicated genes, there is no one-to-one correspondence between genomes and the final encodings of multisets of genes, adjacencies, and telomeres. Once we have the binary sequences and transition parameters, we can reconstruct a phylogeny using maximum likelihood. Of the many implementations of this method, we chose RAxML [23] for its speed and its dedicated handling of binary sequences.

### Bootstrap support

A distinct advantage of using sequence encoding is the ability to use the bootstrap method to assess the robustness of the inferred phylogeny. Doing so with gene-order data is not possible, because a chromosome with *n* distinct genes presents a single character (the ordering) with 2^*n*^×*n*! possible states (the first term is for the strandedness of each gene and the second for the possible permutations in the ordering). This single character is equivalent to an alignment with a single column, albeit one where each character can take any of a huge number of states—we cannot meaningfully resample a single character. The binary encoding effectively maps this single character into a high-dimensional binary vector, so that the standard phylogenetic bootstrap [24] can be used. While the evolution of a specific adjacency depends directly on several others, independence can be assumed if, once an adjacency is broken during evolution, it is not formed again—an analog of Dollo parsimony, but one that is very likely in rearrangement data due to the enormous state space [25].

### Ancestral inference

Using the phylogeny thus computed, we then proceed to solve the SPP, now following the approach of Hu *et al.* [21]. The first step involves the estimation of ancestral gene contents from the contents of the input genomes. Our inference of ancestral contents relies on viewing genes and adjacencies as independent binary characters, as described for the encoding. Whether or not an ancestral genome contains a gene or an adjacency is determined by the conditional probability of the presence state of the gene or the adjacency, computed by the marginal probabilistic reconstruction method suggested by Yang *et al.* [26]. If such probability is larger than 50%, we conclude that the gene belongs to the genome. We extend this approach to compute the probability of observing each adjacency. We then reduce the adjacency assembly problem for any given ancestral genome to an instance of the Travelling Salesperson Problem (TSP), by representing genes as vertices and adjacencies as edges, and finally solve the TSP by using Concorde [27].

## Results and discussion

**Figure 1:**
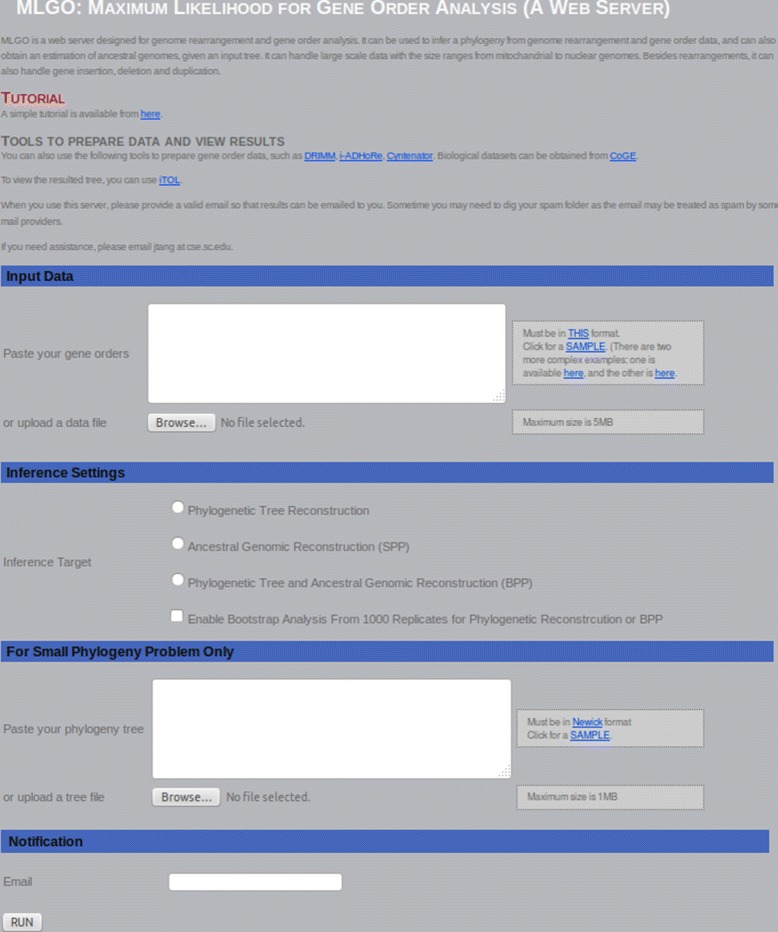
**The screen shot of the web interface for** MLGO **.**

MLGO is written in C++ and Perl as a web tool. Figure 1 shows the screen shot of the web interface for MLGO. The input format of the dataset is that used by GRAPPA and MGR: FASTA-like headers for the names of the genomes (> followed by an alphanumeric sequence followed by a newline), each chromosome represented by a signed permutation of integers ending with a $ symbol and a newline character. Phylogenies are output as trees in Newick format.

**Figure 2:**
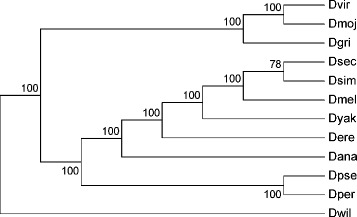
**The consensus phylogeny of 12 drosophila genomes with bootstrap support values from 100 replicates.**

**Figure 3:**
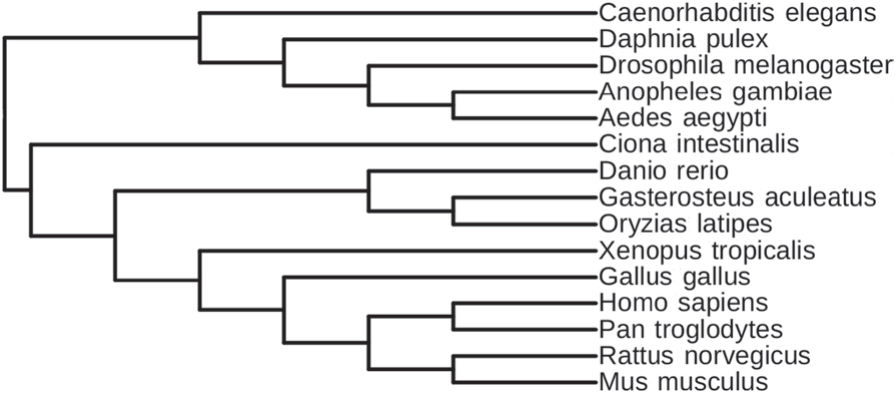
**The reconstructed phylogeny of 15 Metazoan genomes.**

We used the genomes of 12 fully sequenced drosophila species to demonstrate the performance of MLGO. Figure 2 shows the consensus phylogeny reconstructed by MLGO with the bootstrap support values obtained using 100 replicates. Compared to the study using sequence data published by Clark *et al.* [28], all major groups in those 12 drosophila genomes were correctly identified with strong support (bootstrap value >90), except for one median support at the bipartition between *D. simulans*, *D. sechellia* and the rest. The total running time for reconstructing the phylogeny of 12 drosophila species is less than 1 minute, while ancestral reconstruction adds less than 30 minutes. We also tested the performance of MLGO on 15 Metazoan genomes from the eGOB (Eukaryotic Gene Order Browser) database [29], and the reconstructed phylogeny tree shown in Figure 3 is perfectly supported from existing studies [30,31].

## Conclusion

As whole genomes are sequenced at increasing rates, using gene-order data for phylogenetic analyses and ancestral reconstruction is attracting increasing interest, especially coupled with the recent advances in identifying conserved synteny blocks among multiple species [32–34].

MLGO (Maximum Likelihood for Gene-Order Analysis) is the first web tool for likelihood-based inference of both the phylogeny and ancestral genomes. It provides fast and scalable analyses with bootstrap support of large-scale genomic changes including not only rearrangements but also gene insertions, deletions and duplications.

## Availability and requirements

The web tool is available from http://www.geneorder.org/server.php.**Project name:** MLGO**Project home page:**http://www.geneorder.org/server.php**Operating system(s):** Platform independent**Programming language:** Perl**Other requirements:** None**License:** GNU**Restrictions for use by non-academics:** None

## Endnote

^a^ We use the term “gene” as this is in fact a common form of syntenic blocks, but other kinds of markers could be used.
